# Knowledge of obstetric danger signs among child bearing age women in Goba district, Ethiopia: a cross-sectional study

**DOI:** 10.1186/s12884-015-0508-1

**Published:** 2015-03-29

**Authors:** Daniel Bogale, Desalegn Markos

**Affiliations:** Department of Public Health, College of Medicine and Health Sciences, Madawalabu University, Bale Goba, Ethiopia; Department of Nursing, College of Medicine and Health Sciences, Madawalabu University, Bale Goba, Ethiopia

**Keywords:** Obstetric danger signs, Pregnancy, Child birth, Postpartum, Goba district, Ethiopia

## Abstract

**Background:**

Awareness of the danger signs of obstetric complications is the essential first step in accepting appropriate and timely referral to obstetric and newborn care. Ethiopia is a country where maternal morbidity and mortality is high and little is known about knowledge level of reproductive age women on obstetric danger signs. The aim of the study was to assess knowledge of obstetric danger signs among mothers delivered in the last 12 months in Goba district, Ethiopia.

**Methods:**

A community based cross-sectional study was conducted in Goba district. The study included 562 recently delivered women from 9 kebeles (the smallest administrative unit). A safe motherhood questionnaire developed by the Maternal and Neonatal Program of JHPIEGO, an affiliate of John Hopkins University was used to collect data. Binary and multiple logistic regressions were done to explore factors determining maternal knowledge on obstetric danger signs. Variables having P-value of less than or equal to 0.05 on binary logistic regression were the candidate for multiple logistic regressions. Statistical significance was declared at P < 0.05.

**Result:**

One hundred seventy nine (31.9%), 152 (27%) and 124 (22.1%) of study participants knew at least three key danger signs during pregnancy, delivery and postpartum period, respectively. As compared to women who did not attended Anti Natal Care service during their pregnancy, those who attend ANC were 2.56 times and 2.54 times more likely to know obstetric danger signs during pregnancy and child birth (AOR = 2.56 and 95% CI: 1.24-5.25) and (AOR = 2.54 and 95% CI: 1.14-5.66), respectively.

**Conclusion:**

This study showed low level of knowledge of obstetric danger signs during pregnancy, child birth and postpartum period among women in Goba district. This indicates the large proportions of pregnant women who do not have the knowledge are likely to delay in deciding to seek care. ANC follow up was a significant factor for knowledge about obstetric danger signs occurring during pregnancy and child birth. Every woman should be made aware of the likelihood of complications during pregnancy, childbirth/labour and the postpartum periods.

## Background

Maternal mortality remains a public health challenge worldwide, and the global maternal mortality ratio of 342, 900/100,000 live births annually is still unacceptably high [1]. Similarly, maternal mortality is a serious public health problem in Ethiopia. It is estimated that there are 676 maternal deaths per 100,000 live births in Ethiopia [2]. These deaths arise from pregnancy, childbirth or postpartum complications [1].

The commonest danger signs during pregnancy include severe vaginal bleeding, swollen hands/face and blurred vision. Key danger signs during labor and childbirth include severe vaginal bleeding, pro-longed labor, convulsions, and retained placenta. Danger signs during the postpartum period include severe bleeding following childbirth, loss of consciousness after childbirth, and fever [3,4]. Recognizing these danger signs for pregnancy related complications and what to do if they arise would significantly increase the capacities of women, their partners and their families to remain healthy, to take appropriate steps to ensure a safe birth and to seek timely skilled care in emergencies [5]. The key elements of the birth plan package include recognition of danger signs, a plan for a birth attendant, a plan for the place of delivery, and saving money for transport or other costs in case the need arises [6]. Every pregnant woman faces the risk of sudden, unpredictable complications that could end in death or injury to herself or to her infant [7].

The national reproductive strategy of Ethiopia has given emphasis to maternal and newborn health so as to reduce the high maternal and neonatal mortality. The strategy focuses on the need to empower women, men, families and communities to recognize pregnancy related risks, and to take responsibility for developing and implementing appropriate response to them. One of the targets in the strategies is to ensure that 80% of all families recognize at least three danger signs associated with pregnancy related complications by 2010 in areas where health extension program is fully implemented [8]. Despite the fact that emphasis is given by the national strategy to raise knowledge of obstetric danger signs little is known about the current level of knowledge and the influencing factors in Ethiopia [9].

Knowledge of danger signs of obstetric complications during pregnancy, labour and postnatal period is the first essential step for appropriate and timely referral. It is also strategy aimed at enhancing the utilization of skilled care during low risk births and emergency obstetric care in complicated cases in low income countries [3]. The majority of pregnant women and their families do not know how to recognize the danger signs of complications. When complications occur, the unprepared family will waste a great deal of time in recognizing the problem, getting organized, getting money, finding transport and reaching the appropriate referral facility [10]. Raising awareness of pregnant women on the danger signs would improve early detection of problems and reduces the delay in deciding to seek obstetric care [3,4].

A community based cross-sectional study conducted in Tanzania showed that about half of the study subjects knew at least one obstetric danger sign [11]. Descriptive cross- sectional study carried out among antenatal care clients at Kenya National Hospital showed that 27.9% of the study respondents were not informed about danger signs in pregnancy. According to this study, Haemorrhage was the most known danger sign in pregnancy mentioned by 64.2% of the respondents, followed by reduced foetal movement which was mentioned by 20.6% of the respondents [12]. Another descriptive survey conducted in Southern Nigeria reveals that, about half of the respondents (51.9%) considered bleeding, about a third considered convulsions (37.8%) and loss of consciousness (33.2%) as danger signs in pregnancy [13]. The percentage of women who knew at least one danger sign related to pregnancy was 26%, in relation to delivery was 23%, and to the period after delivery was 40% [11]. Therefore, this study was undertaken to explore the level of knowledge about obstetric danger signs among recently delivered women in Goba district, Oromia Region, Ethiopia.

## Methods

### Study design and setting

We carried out a community based cross sectional study in Goba district, Bale zone Ethiopia during April, 2013. Currently, the district has 24 rural and 2 urban kebeles. The estimated total number of women of reproductive age (15-45 years) and pregnant women in the district is 16,277 and 2725, respectively. A total of 43 health institutions were available in the district: 13 in Goba town including 2 government health facilities (1 hospital and 1 health center) and 11 private health facilities (6 clinics and 5 pharmacies), and 30 outside Goba town including 28 government health facilities (3 health centers, 24 health posts and 1 clinics) and 2 private clinics [14].

Mothers who had been living for at least 6 months in Goba district and who had given birth within the last 12 months prior to the data collection, irrespective of birth outcome, were the source population of this study.

### Sample size determination

Using the formula for single population proportions, we made the following assumptions: proportion of mothers knowing obstetric danger signs during pregnancy to be 23.8% [5], confidence level 95%, absolute precision +/-5%, design effect 2, and non-response rate 5%. The required sample size was thus 580.

### Sampling procedure

All kebeles of Goba district were stratified into urban (n = 2) and rural ones (n = 24). Roughly 1/3 of the kebeles in each stratum, i.e. one urban and eight rural kebeles, were selected by simple random sampling. Census was carried out in the selected kebeles to identify mothers who gave birth in the last one year prior to the survey. Then, the total sample size (n = 580) was allocated proportionally on each kebele based on the number of mothers delivered during the last 12 months prior to the data collection of this study. Finally, systematic sampling was employed to select the study subjects in each kebele until the desired numbers of sample was obtained. To select the first house hold in each kebele, a starting point was identified at the centre of each kebele with the help of health extension workers (actors in an innovative community based health care delivery system to accelerate expansion of primary health care coverage program) assigned in that kebele.

### Operational and term definitions

Knowledge of obstetric complication(s): Any symptom of obstetric complication(s) reported by woman which may occur in women during pregnancy, delivery or within 6 weeks after delivery.

Knowledgeable on key danger signs of pregnancy: In this research a woman was considered as knowledgeable if she can mention at least three key danger signs for pregnancy.

Knowledgeable on key danger signs of labor/childbirth: In this research a woman was considered as knowledgeable if she can mention at least three key danger signs for Labor/childbirth spontaneously or after prompting.

Knowledgeable on key danger signs of post partum: In this research a woman was considered Knowledgeable if she can mention at least the three key danger signs for post partum spontaneously or after prompting.

### Data collection procedure

A safe motherhood questionnaire developed by the Maternal and Neonatal Program of JHPIEGO, an affiliate of John Hopkins University was used after adoption to actual set up [3]. The questionnaire was translated from English to local language. The questionnaire was pre-tested on 5% of the total sample size in Robe town and a necessary adjustment was made before use for data collection. Eight diploma Nurses who were fluent in speaking local language were involved in the data collection. Two Bachelor of Science degree (BSc) holder health professionals were recruited as supervisors.

### Data quality control

After pre-testing the questionnaire, Cronbatch’s Alpha was calculated by using SPSS window version 16.0 to test internal consistency (reliability) of the item and Cronbatch’s Alpha greater than 0.7 was considered as reliable. On the top of this, content validity was cross checked by another maternal and/or reproductive health expert. Data collectors and supervisors were trained for three days (including practical sessions) on the study instrument and data collection procedure. The principal investigator and the supervisors checked the collected data for completeness and corrective measures was taken accordingly.

### Data processing and analysis

The data was checked for completeness and consistencies, then it was coded and entered in to computer using statistical package for social sciences (SPSS) windows version 16.0. and further clearance was made after entry using this software. The level of knowledge on key danger signs of obstetric complication during pregnancy, child birth and during post partum period was described and in this study a woman was considered knowledgeable when she mention at least three recognized danger signs for each of the aforementioned period. Additionally, binary logistic regression was determined to see the independent effect of an independent variable during pregnancy, child birth and during post partum period. Furthermore, multiple logistic regressions were carried out to examine the existence of relationship between the aforementioned outcome variables and selected determinant factors. Variables significant in the bivariate analysis were then entered into a multiple logistic regression analysis. The associations between knowledge on key danger signs of obstetric complication during the three period (pregnancy, child birth and during post partum) and each independent variable were estimated by odds ratio (OR) and 95% confidence interval (CI). A CI was considered statistically significant when the interval between the upper and lower values did not include one.

### Ethical consideration

The proposal was approved by Ethical Review Committee of College of Medicine and Health sciences of Madawalabu University. Furthermore, letter of permission was obtained from Bale Zone health department and from Goba Woreda administrative and health offices. Verbal consents was obtained from the study subjects after explaining the study objectives and procedures and their right to refuse not to participate in the study any time they want was assured. For this very purpose, a one page consent letter was attached to the cover page of each questionnaire stating about the general objective of the study and issues of confidentiality which was discussed by the data collectors before proceeding with the interview.

## Results

### Socio demographic characteristics

Out of the total 580 mothers who were planned for the study, 562 were successfully interviewed yielding the response rate of 97%. The mean age of the study subjects was 26.6 (SD ± 5.96). Muslim and Orthodox Tewahido were found as a dominant religion each accounting 49.1%. Most participants, 266 (47.3%), reported that they were attended up to primary school. Four hundred ten (73%) of the respondents were ethnically Oromo, followed by Amhara; 139 (24.7%). The vast majority, (85.2%), of respondent were house wife. Regarding marital status of the respondents, 531(94.5%) were married. The mean family size and mean of the estimated income of the participants were 5.01 (SD ± 2.05) and 1267.86 (SD ± 1298.729), respectively. Nearly half, (47.3%), of the study subjects’ husband were educated to the primary level (Table 1).

**Table 1:** **Socio-demographic characteristics of the respondents in Goba district, Ethiopia, April 2013**

**Variable**	**Frequency**	**Percent**
**Residence**		
Urban	116	20.6
Rural	446	79.4
**Age**		
≤20	94	16.7
21-25	190	33.8
26-30	164	29.2
>30	114	20.3
**Religion**		
Muslim	276	49.1
Orthodox	276	49.1
Protestant	7	1.2
Other*	3	0.5
**Marital status**		
Married/in Union	531	94.5
Single	14	2.1
Widowed	8	1.4
Divorced	6	1.1
Separated	3	0.5
**Ethnicity**		
Oromo	410	73
Amhara	139	24.7
Other**	13	2.3
**Educational level**		
Cannot read and write	161	28.6
Read and write	16	2.8
Primary	266	47.3
Secondary and above	119	21.2
**Occupation**		
Housewife	479	85.2
Gov’t. employee	21	3.7
Private employee	14	2.5
Merchant	42	7.7
Other***	6	1.1
**Total family income**		
≤100	12	2.1
101-300	60	10.7
≤301	487	87.1
**Husbands educational level**		
Cannot read and write	83	15.3
Read and write	31	5.7
Primary	271	48.2
Secondary and above	159	28.3
**Husbands’ occupation**		
Farming	336	61.8
Gov’t. employee	76	14
Private employee	80	14.7
Merchant	52	9.6

*include Catholic and Wakefeta, **include Tigrai, Gamo, Welayita, Gurage & ***include daily laborer.

### Knowledge on danger signs during pregnancy, child birth and postpartum period

About sixty eight percent, 383 (68.1%), mothers were found to be not knowledgeable and 179 (31.9%) mothers were knowledgeable about danger sign during pregnancy. Similarly, 410 (73%), of women under the study were found to be not knowledgeable and only 152 (27%) mothers were knowledgeable about danger sign during labour. Additionally, about 438 (77.9%) study participant were found to be not knowledgeable and 124 (22.1%) were knowledgeable about danger sign during postpartum period.

Severe vaginal bleeding was the most frequently mentioned complication by women during pregnancy 191(71.3%), and postpartum period 150(76.5%) (Table 2).

**Table 2:** **Knowledge of mothers about obstetric danger signs during pregnancy and post partum period in Goba district, Ethiopia, April 2013**

**Danger signs**	**Awareness of danger signs during**			
	**Pregnancy**		**Postpartum**	
	**N**	**%**	**N**	**%**
Vaginal bleeding	191	71.3	150	76.5
Severe headache	144	53.7	78	39.8
Blurred vision	37	13.8	11	5.6
High fever	50	18.7	56	28.6
Loss of consciousness	50	18.7	68	34.7
Convulsions	27	10.1	20	10.2
Swollen hands/face	123	45.9	56	28.6
Difficulty in breathing	17	6.3	17	8.7
Severe weakness	119	44.4	90	45.9
Severe abdominal pain	46	17.2	NA	NA
Accelerated/reduced fetal movement	47	17.5	NA	NA
Water breaks without labor	65	24.3	NA	NA
Malodorous vaginal discharge	NA	NA	46	23.5
Other	7	2.6	6	3.1

*NA: imply danger sign was not assessed for that period.*

**Figure 1:**
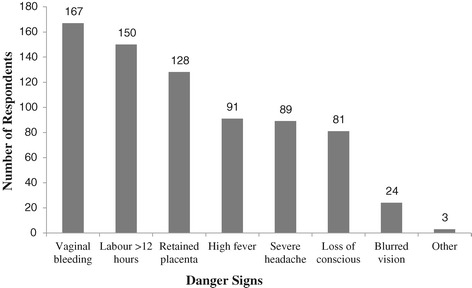
**Obstetric danger signs known by the study participants during delivery in Goba district, Ethiopia, April 2013.**

Vaginal bleeding, prolonged labour and retained placenta were the most frequently indentified obstetric danger signs during labour by respondents (Figure 1).

### Factors affecting knowledge of obstetric danger signs

The independent variable residential place was statistically significant for the knowledge of obstetric knowledge of women occurring during child birth; as compared to a woman living in rural area, a woman living in urban area was about two times more likely to know danger signs occurring during delivery (AOR = 2.2 and 95% CI = 1.26-3.97). However, it didn’t show statistical association with the knowledge of woman about the danger signs during pregnancy and post partum period. Mothers who can read and write were more likely to know obstetric danger signs of child birth and post partum period than those who cannot read and write (AOR = 4.6 and 95% CI = 1.57-13.73) and (AOR = 3.0 and 95% CI = 1.07-8.70), respectively. Government employee mothers were more likely to know obstetric danger signs of child birth than housewife mothers (AOR = 3.6 and 95% CI = 1.14-11.75). ANC service utilization was another statistically significant independent variable for knowledge about obstetric danger signs during pregnancy and child birth (Table 3).

**Table 3:** **Factors associated with knowledge of key obstetric danger sign during pregnancy, labour and postpartum in Goba district, Ethiopia, April 2013**

**Characteristics**	**AOR (95% CI) for knowledge about danger signs of pregnancy**	**AOR (95% CI) for knowledge about danger signs during labor**	**AOR (95% CI) for knowledge about danger signs in postnatal period**
**Residence**			
Urban	1.32 (0.76, 2.30)	2.2 (1.26, 3.97)*	1.6 (0.89,2.94)
Rural	1	1	1
**Age**			
<20	1		
20-24	0.61 (0.32, 1.17)		
25-29	0.70 (0.38, 1.28)	**	**
30-34	0.31 (0.16, 0.74)		
> = 35	0.90 (0.45, 1.82)		
**Marital status**			
In marital union	**	**	0.06 (0.01, 12.03)
Not in marital union			1
**Educational status of mother**			
Cannot read and write	1	1	1
Read and write	0.82 (0.22, 3.00)	1.3 (0.35, 4.87)	0.6 (0.13, 3.29)
Primary	1.13 (0.65, 1.96)	1.5 (0.82, 2.76)	1.2 (0.67, 2.35)
Secondary and above	1.63 (0.78, 3.42)	2.4 (1.07, 5.41)*	1.2 (0.55, 2.91)
**Educational status of husband**			
Cannot read and write	1	1	1
Read and write	5.71 (2.07, 15.76)*	4.6 (1.57, 13.73)*	3.0 (1.07, 8.70)*
Primary	1.66 (0.80, 3.45)	1.4 (0.65, 3.22)	1.0 (0.50, 2.40)
Secondary and above	2.40 (1.01, 5.69)*	1.6 (0.65, 4.18)	1.6 (0.66, 4.18)
**Occupation of mother**			
Housewife	1	1	1
Gov’t. employee	1.51 (0.51, 4.45)	3.6 (1.14, 11.75)*	2.1 (0.70, 6.38)
Private employee	0.83 (0.21, 3.16)	0.7 (0.16, 3.10)	0.6 (0.12, 3.22)
Merchant	0.71 (0.31, 1.59)	1.7 (0.46, 2.45)	1.2 (0.52, 2.94)
Daily laborer	0.52 (0.41, 6.61)	4.0 (0.29, 55. 56)	0.9 (0.06, 11.84)
**Occupation of Husband**			
Farmer	1	1	1
Gov’t Employee	1.24 (0.60, 2.55)	1.0 (0.50, 2.30)	1.2 (0.57, 2.78)
Merchants	1.70 (0.90, 3.23)	1.3 (0.70, 2.73)	0.9 (0.44, 1.88)
Others	1.42 (0.66, 3.02)	1.20 (0.54, 2.70)	0.9 (0.40, 2.19)
**Distance from nearest HF**			
<15 minutes	0.62 (0.38, 1.02)	0.6 (0.37, 1.10)	0.5 (0.32, 0.97)*
15-30minutes	1.68 (1.04, 2.71)*	2.09 (1.26, 3.44)*	1.4 (0.86, 2.40)
>30 minutes	1	1	1
**ANC follow up**			
Yes	2.56 (1.24, 5.25)*	2.54 (1.14, 5.66)*	**
No	1	1	
**Number of pregnancy**			
One	**	1	1
Two –Four		0.82 (0.49, 1.36)	0.7 (0.44, 1.23)
> = Five		0.87 (0.47, 1.63)	0.4 (0.25,0.95)*
**Place of delivery**			
Home	1	1	1
Health institution	0.80 (0.50, 1.28)	0.6 (0.40, 1.11)	0.9 (0.54, 1.52)

*Adjusted for all other variables in the table.

**Indicating the p-value of the variable considered in that specific cell was greater than 0.05 which was not a candidate for multiple logistic regression.

## Discussion

Knowledge of danger signs of obstetric complications during pregnancy, labour and postnatal period is the first essential step for appropriate and timely referral. About one third, 179 (31.9%) of mothers were knowledgeable about danger sign during pregnancy. This finding is consistent with the study conducted in Aleta Wondo in which 30.9% of respondents mentioned at least two danger signs of pregnancy [9]. Out of the women under the study, 152 (27%) were knowledgeable about danger signs during labour. Additionally, 124 (22.1%) were knowledgeable about danger signs during postpartum period which is not consistent with similar study in which 37.7% of women know at least two danger signs during post partum period [9]. The previous study considered a mother was knowledgeable if she answered at least two danger signs happening during post partum period where as in the current study at least three danger signs should be answered by a mother to say she is knowledgeable which might be the reason for this discrepancy. The finding of this study was higher than the study conducted in rural Tanzania in which the percentage of women who knew at least three danger sign related to pregnancy was 6.9%, in relation to delivery 1.3%, and to the period after delivery 3.3% [11]. This difference could be resulted from the variation in educational level of respondents and accessibility of information in these two study settings. Similarly, it is higher than the findings from study conducted in rural Uganda in which 19% mothers had knowledge of 3 or more key danger signs during the three periods [15]. These differences in knowledge level could again be due to a difference in socio-demographic, cultural, and health interventions as well as methodological difference.

In this study, Vaginal bleeding was the most recognized obstetric danger sign all during pregnancy, 191 (71.3%), labour, 167 (63.5%) and postpartum period, 150 (76.5%). It is in line with other similar studies conducted in different countries [9,11,15,16]. This could be an indication of awareness by women that bleeding is the main and fastest cause of maternal mortality.

Retained placenta, 128 (48.7%) and labour lasting greater than 12 hours, 150 (57%) were the two major obstetric danger signs mentioned by the respondents during child birth. These findings were also in line with the study conducted in Aleta Wondo where knowledge of retained placenta was (51.4%) and labour lasting greater than 12 hours was (43.2%) [9]. Somewhat, it was higher as compared to the study conducted in Uganda where awareness of respondents about retained placenta as a danger sign during child birth was (35.1%) and labour lasting greater than 12 hours (18.3%) [15]. This discrepancy might be due to the deference in socio-cultural and socio-economic variations in these study settings. In addition to this, this study included both rural and urban settings where as the study conducted in Uganda considered only rural settings.

Women who live in urban area were 2.2 times more likely to mention at least three danger signs during post partum period as compared with rural counterparts. This variable was also found to have a significant association with mentioning at least two danger signs during pregnancy, child birth and postpartum period in other study [9]. This could be due to the fact that urban residents have better access to health information and maternal health services as compared with rural counterparts.

As compared to housewife, government employee women were about four times more likely to mention at least three danger signs during labour. This could be explained by the fact that, a woman who has her own income might be autonomous in seeking better health care than house wife who has no their own income.

One of the most important functions of antenatal care is to offer the woman advice and information about birth preparedness, danger signs of obstetric complication in pregnancy, child birth and in the post partum period and emergency preparedness [12]. In this study, ANC follow up was found to have statistically significant association with maternal knowledge about obstetric danger signs during pregnancy and child birth. However, it didn’t show statistically significant association for the knowledge about danger signs occurring during postpartum period. This could be related with the content and quality of ANC services given in health care settings.

As a strength, this study tried to minimize selection bias by employing community based study with probability sampling method. Additionally, recall bias was attempted to be reduced by involving women who have given birth in the last 12 months preceding the study. On the other hand, its limitation is associated with not ascribing the direction of causations to the relationships found in the study because of the nature of cross sectional study design.

## Conclusion

This study showed low level of knowledge of obstetric danger signs during pregnancy, child birth and postpartum period among women in Goba district. This indicates the large proportions of pregnant women who do not have the knowledge about obstetric danger signs may be delay in deciding to seek care. The most frequently cited obstetric complication of pregnancy, child birth and postpartum was vaginal bleeding. ANC follow up was a significant factor for knowledge about obstetric danger signs occurring during pregnancy and child birth. Therefore, every woman should be made aware of the likelihood of complications during pregnancy, childbirth/labour and the postpartum periods. Interventions targeting improvement of maternal health need including the quality of information offered to pregnant women during ANC follow up is recommended.

## References

[1] Kakaire O, Kaye DK, Osinde MO: Male involvement in birth preparedness and complication readiness for emergency obstetric referrals in rural Uganda. Reprod Health. 2011, 8(12). 10.1186/1742-4755-8-12 PMC3118172 21548976

[2] ECSA, ICF. Ethiopian Demographic and Health Survey 2011. Edited by International. CSAaI. Addis Ababa, Ethiopia and Calverton, Maryland, USA; 2011.

[3] JHPIEGO (2004). Maternal and Neonatal Health Pro-gram. Birth Preparedness and Complication Readiness: A Matrix of Shared Responsibilities.

[4] Thaddeus S, Maine D (1994). Too far to walk: maternal mortality in context. Soc Sci Med.

[5] WHO (2006). Standards for Maternal and Neonatal Care: Birth and emergency preparedness in antenatal care: Department of Making Pregnancy Safer (MPS).

[6] JHPIEGO (2001). Maternal and Neonatal health (MNH) Program, Birth preparedness and complication readiness. A matrix of shared responsibilities. Maternal and Neonatal Health.

[7] Hiluf M, Fantahun M (2007). Birth Preparedness and Complication Readiness among women in Adigrat town, north Ethiopia. Ethiop J Health Dev.

[8] Federal Democratic Republic of Ethiopia, Ministry of Health (2006). National Reproductive Strategy, 2006-2015.

[9] Hailu M, Gebremariam A, Alemseged F (2010). Knowledge about obstetric danger signs among pregnant women in Aleta Wondo district, Sidama Zone, Southern Ethiopia. Ethiop J Health Sci.

[10] Moore M, Copeland R, Chege I, Pido D, Griffiths M (2002). A behavior change’s approach to investigating factors influencing women’s use of skilled care in Homa Bay District Kenya.

[11] Pembe AB, Urassa DP, Carlstedt A, Lindmark G, Nyström L, Darj E: Rural Tanzanian women’s awareness of danger signs of obstetriccomplications. BMC Pregnancy Childbirth. 2009, 9(12). 10.1186/1471-2393-9-12 PMC2667432 19323836

[12] Mutiso SM, Qureshi Z, Kinuthia J (2008). Birth preparedness among antenatal clients. East Afr Med J.

[13] Iliyasu Z, Abubakar IS, Galadanci HS, Aliyu MH (2010). Birth Preparedness, Complication Readiness and Fathers’ Participation in Maternity Care in a Northern Nigerian Community. Afr J Reprod Health.

[14] Nigatu D: Women’s autonomy regarding their own and children’s Health care utilization and associated factors in Goba Woreda, Bale zone, south east Ethiopia. Unpublished. Public health; 2011

[15] Kabakyenga JK, Östergren P-O, Turyakira E, Pettersson KO: Knowledge of obstetric danger signs and birth preparedness practices among women in rural Uganda. Reprod Health. 2011, 8(33). 10.1186/1742-4755-8-33 PMC3231972 22087791

[16] Udofia EA, Obed SA, Calys-Tagoe BNL, Nimo KP (2013). Birth and emergency planning: a cross sectional survey of postnatal women at Korle Bu teaching hospital, Accra, Ghana. Afr J Reprod Health.

